# Peripubertal pregnancies and associations with health and developmental outcomes in offspring during late adolescence and adulthood: sibling-comparison register study

**DOI:** 10.1093/ije/dyag153

**Published:** 2026-08-27

**Authors:** Vasudha Ahuja, Ayako Hiyoshi, Helena Backman, David Simmons, Scott Montgomery

**Affiliations:** Clinical Epidemiology and Biostatistics, School of Medical Sciences, Faculty of Medicine and Health, Örebro University, Örebro, Sweden; Clinical Epidemiology and Biostatistics, School of Medical Sciences, Faculty of Medicine and Health, Örebro University, Örebro, Sweden; Department of Public Health Sciences, Stockholm University, Stockholm, Sweden; Department of Obstetrics and Gynaecology, Faculty of Medicine and Health, Örebro University, Örebro, Sweden; Macarthur Clinical School, School of Medicine, Western Sydney University, Campbelltown, Australia; School of Medical Science, Faculty of Medicine and Health, Örebro University, Örebro, Sweden; Clinical Epidemiology and Biostatistics, School of Medical Sciences, Faculty of Medicine and Health, Örebro University, Örebro, Sweden; Clinical Epidemiology Division, Department of Medicine, Solna, Karolinska Institutet, Stockholm, Sweden; Department of Epidemiology and Public Health, University College of London, London, United Kingdom

**Keywords:** peripubertal pregnancies, teenage pregnancies, adult offspring outcomes, metabolic outcomes, developmental outcomes, register study, sibling-comparison study

## Abstract

**Background:**

We examined the association of peripubertal pregnancies with long-term health and developmental outcomes in adult offspring and strengthened causal inference using sibling-comparison methods.

**Methods:**

Using linked Swedish register data from 1964 to 2018, we conducted a cohort study to estimate associations of pregnancies before age 16 years compared with pregnancies between ages 16 and 25 years, with body mass index (BMI), systolic blood pressure (SBP), cognitive function, and stress resilience among 943 444 male offspring aged 17–21 years in the Military Conscription Register. We further assessed associations with type 2 diabetes (T2D), depression, and anxiety among 2 461 510 male and female offspring aged 18–73 years using population-wide registers. Linear, multinomial, and survival models were used. Sibling fixed-effect models were used to account for unmeasured confounding shared among siblings.

**Results:**

Offspring from pregnancies before age 16 years compared with pregnancies between ages 16 and 25 years had lower SBP (β − 1.12; 95% confidence interval [CI] − 1.97 to −0.26), poorer cognitive function (β  − 0.54; 95% CI −0.67 to −0.40), and poorer stress resilience (β −0.24; 95% CI −0.38, −0.10). There was no increased risk of being underweight or overweight compared with normal weight. There was a lower risk of anxiety (hazard ratio 0.87; 95% CI 0.80 to 0.95) and no increased risk of T2D or depression. Sibling-comparison analyses attenuated deleterious associations to null or reversed them and amplified associations with markers of better health.

**Conclusion:**

Pregnancies before age 16 years may not be causally associated with adverse long-term health and developmental outcomes in adolescent and adult offspring. The adverse associations in conventional analyses appear attributable to shared familial characteristics.

Key Messages

- We examined associations of pregnancies before age 16 years, likely during the peripubertal period, with health and developmental outcomes in offspring aged 17–83 years.
- We found that any apparently harmful associations with having a mother who was pregnant before age 16 years were eliminated or reversed by comparing outcomes between siblings, which accounts for unmeasured shared family characteristics.
- Pregnancies before 16 years may not be causally associated with adverse health or developmental outcomes among offspring in late adolescence or adulthood.

## Introduction

Offspring of women who were teenagers when pregnant (aged ≤19 years) have been reported to experience poor health and developmental outcomes [1–10]. In developed countries, teenage pregnancies are more often among those from a socioeconomically disadvantaged background than pregnancies at older ages [11]. Previous work demonstrated that the magnitude of this association attenuated after adjusting for maternal socioeconomic position [1, 2]. The key sources of residual confounding include stable maternal characteristics that predispose to both very early pregnancies and offspring outcomes [12]. These include cognitive, behavioural, and health-related traits [12]. Sibling comparisons provide a particularly appropriate design for examining these associations [12]. Accordingly, these have been employed to strengthen causal inference by controlling for shared genetic and environmental factors within families [3–8, 13].

Some sibling-comparison studies have previously investigated associations of teenage pregnancies with educational attainment and labour market outcomes in offspring [6–9]. To the best of our knowledge, none have investigated metabolic health outcomes extending into adulthood [1–8]. Moreover, prior research has typically defined teenage pregnancies broadly (age ≤19 years), potentially masking heterogeneity within adolescence [1–10]. In contrast, the present study focuses on pregnancies before 16 years of age. This period corresponds to early adolescence and the peripubertal period for many girls [14–17]. This is characterized by a marked increase in insulin resistance, which peaks during mid-puberty, corresponding approximately to Tanner stages 3–4 and ages 11–15 years [14–17].

Pregnancies occurring during this period may therefore be superimposed on an already insulin-resistant metabolic state, with additional insulin resistance induced by placental hormones [18]. Exposure to such an intrauterine metabolic environment may result in adverse foetal programming, with lasting consequences for cardiometabolic regulation in the offspring [19, 20]. In addition to biological pathways, early adolescent motherhood may influence offspring outcomes through psychosocial mechanisms. These mothers may be less prepared for parenting, experience greater psychosocial stress, and face persistent socioeconomic disadvantage [9, 10, 21]. These factors may affect caregiving quality and the developmental environment during childhood [9, 10, 21].

Accordingly, we examined associations between pregnancies before 16 years and offspring cardiometabolic outcomes, including systolic blood pressure (SBP), body mass index (BMI), and type 2 diabetes (T2D), assessed from late adolescence to adulthood using a longitudinal sibling-comparison design based on Swedish register data. As secondary outcomes, we examined cognitive function and stress resilience measured at conscription as early-life markers, and depression and anxiety diagnosed in adulthood as later-life outcomes. These share biological and behavioural pathways with cardiometabolic disease [22–27] and provide a broader life-course perspective on the long-term implications of very early maternal age at birth.

## Methods

### Study design and data sources

We conducted a cohort study by linking prospectively recorded data (1964–2018) from multiple Swedish national registers using pseudonymized individual identity numbers. Maternal characteristics were obtained from the Medical Birth Register (MBR) [28] and family relationships from the Multi-Generation Register (MGR) [29]. Demographic information was retrieved from the Total Population Register (TPR) [30]. Socioeconomic indicators were obtained from the decennial population and housing censuses (1960–90) and the Longitudinal Database of Health, Insurance and Labour Market Studies (LISA) [31, 32]. Data on physical and mental assessments at military conscription assessment were obtained from the Military Conscription Register (MCR) [33]. Information on inpatient care (from 1964) and outpatient care (from 2001) was extracted from the National Patient Register (NPR) [34]. Data on cause of death were retrieved from the National Cause of Death Register (NCDR) [35], and prescribed medications were retrieved from the National Prescribed Drug Register (NPDR) [36].

### Study sample

We used two study populations. Cohort I comprised 934 444 late adolescent and young adult males aged 17–21 years, identified in the MCR. The sibling-comparison sub-cohort consisted of 493 192 offspring born to 223 015 mothers.

Cohort II included 2 461 510 adult males and females identified through the NPR, NCDR, and NPDR. The sibling-comparison sub-cohort consisted of 2 151 783 offspring born to 875 368 mothers. For full details, see supplementary methods.

### Exposure

The biological mother’s age at delivery was derived through record linkage of the TPR and MGR. Maternal age was then categorized as: <16 years, 16–25 years, 26–35 years, and ≥36 years.

### Outcomes

#### Cohort I

BMI and SBP were obtained from the MCR and analysed as early cardiometabolic outcomes in late adolescence. Cognitive function and stress resilience were also obtained from the MCR and included as secondary outcomes reflecting neurodevelopment and stress-regulation. These outcomes are sensitive to foetal and early-life exposures and have been linked to later cardiometabolic and mental health outcomes [20, 22–27]. They may thus provide an insight into potential developmental pathways linking very young maternal age with later disease risk.

#### Cohort II

Diabetes, anxiety, and depression were identified by record linkage to the NPR, NCDR, and NPDR. T2D cases identified in death records were included when listed as an underlying or contributing cause of death. T2D was defined using an age threshold of ≥30 years to distinguish it from type 1 and other forms of diabetes. For full details, see supplementary methods.

### Covariates

In cohort I, when analysing the association of pregnancies <16 years with BMI, SBP, cognitive function, and stress resilience among 17–21 year-old males, conscription age, maternal socioeconomic index (SEI) during childhood (grandparental SEI), and maternal country of birth were included as covariates in the conventional analyses, while conscription age alone was used in sibling-analyses. In cohort II, when analysing the association of pregnancies <16 years with T2D, depression, and anxiety among adult offspring, sex, grandparental SEI, and maternal country of birth were used as covariates in the conventional analysis, whereas sex alone was used in sibling-comparison models. For full definitions, see supplementary methods.

### Statistical analyses

In descriptive analyses, mean (±standard deviation) or median (±interquartile range) and percentages were calculated for continuous and categorical variables. For the multivariable analyses, we performed a complete-case analysis because approximately 48% of observations had missing data for the maternal SEI during childhood. Given the substantial proportion of missing data, we did not perform multiple imputation. In cohort I, we used linear regression for conventional analyses and fixed-effects linear regression for sibling-comparison analyses to examine the associations of pregnancies before 16 (vs 16–25 years) with SBP, cognitive function, and stress resilience. Multinominal logistic regression was utilized for BMI, with and without fixed effects.

In cohort II, person-time was estimated from birth until diagnosis of T2D, depression, or anxiety, death, emigration, or the end of follow-up (31 December 2018), whichever came first. Associations were examined using Cox regression for conventional analyses and stratified Cox regression for sibling-comparison analyses. Proportional hazards assumptions were not violated based on residual plots.

Sibling-comparison analyses control for unmeasured familial environmental and genetic confounding by comparing siblings born to the same mother at different maternal ages (<16 vs ≥16 years). The estimand is within family-effect of maternal age on offspring outcomes. Models were stratified by the biological mother’s identification number and included only families with siblings [37]. Effect estimates were derived from siblings discordant in exposure and, for binary outcomes, also discordant in outcome [38]. Robust sandwich estimators were used to account for familial clustering. Results from sibling-comparison models were compared with conventional population models to assess the extent to which observed associations were explained by shared familial confounding.

### Sensitivity analyses

In sensitivity analyses, we adjusted models for paternal age and subsequently for birth weight and gestational age to assess the robustness of the findings. We further examined whether associations varied by grandparental socioeconomic position, offspring sex (for T2D, depression, and anxiety), and offspring birth cohort to explore potential effect modification. All analyses were performed using R version 4.5.1. For full details, see supplementary methods.

## Results

Tables 1 and 2 present the baseline characteristics of cohorts I and II stratified by maternal age (<16, 16–25, 26–35, and ≥36 years). In cohort I, 1226 (0.13%) and in cohort II, 2758 (0.11%) offspring were of pregnancies before age 16 years. These offspring were more likely to originate from socioeconomically disadvantaged families, to have mothers of foreign origin, and to have lower birth weight.

**Table 1:** Cohort I—based on conscription participation—maternal and offspring characteristics at baseline by maternal age at delivery.

Group	Variable	*N*	<16 *N* = 1226^a^	**16–25 *N* = 418 341** ^a^	**26–35 *N* = 481 479** ^a^	36+ *N *= 42 398^a^	Overall *N* = 943 444^a^
Maternal characteristics	Maternal socioeconomic index during childhood	455 327					
	Medium		121 (15.9%)	46 029 (21.7%)	69 842 (30.1%)	3389 (32.8%)	119 381 (26.2%)
	High		55 (7.2%)	19 215 (9.1%)	25 775 (11.1%)	1454 (14.1%)	46 499 (10.2%)
	Low		460 (60.4%)	115 411 (54.4%)	98 562 (42.5%)	3658 (35.4%)	218 091 (47.9%)
	Agriculture		76 (10.0%)	24 859 (11.7%)	30 540 (13.2%)	1469 (14.2%)	56 944 (12.5%)
	Other		49 (6.4%)	6765 (3.2%)	7234 (3.1%)	364 (3.5%)	14 412 (3.2%)
	(Missing)		465	206 062	249 526	32 064	488 117
	Maternal immigration background	943 444					
	Domestic (two domestic parents)		1077 (87.8%)	376 088 (89.9%)	440 830 (91.6%)	39 338 (92.8%)	857 333 (90.9%)
	Foreign ancestry/foreign-born		149 (12.2%)	42 253 (10.1%)	40 649 (8.4%)	3060 (7.2%)	86 111 (9.1%)
	Paternal age at birth (years)	943 444					
	<16		29 (2.4%)	55 (0.0%)	0 (0.0%)	0 (0.0%)	84 (0.0%)
	16–20		406 (33.1%)	13 024 (3.1%)	180 (0.0%)	3 (0.0%)	13 613 (1.4%)
	20+		254 (20.7%)	257 309 (61.5%)	322 821 (67.0%)	25 759 (60.8%)	606 143 (64.2%)
	Not defined		537 (43.8%)	147 953 (35.4%)	158 478 (32.9%)	16 636 (39.2%)	323 604 (34.3%)
Offspring characteristics	Year of birth	943 444	1969 (1963, 1974)	1972 (1966, 1979)	1978 (1972, 1984)	1984 (1979, 1987)	1976 (1969, 1983)
	Birth weight (kg)	587 105	3.37 (0.59)	3.53 (0.53)	3.61 (0.54)	3.61 (0.58)	3.58 (0.54)
	(Missing)		838	218 998	133 014	3489	356 339
	Gestational age (weeks)	586 612	39.06 (2.44)	39.60 (1.86)	39.58 (1.74)	39.29 (1.83)	39.57 (1.79)
	(Missing)		848	219 450	133 052	3482	356 832

a Mean (standard deviation); number (%).

**Table 2:** Cohort II—based on complete Swedish register data- maternal and offspring characteristics at baseline by maternal age at delivery.

Group	Variable	*N*	<16 *N* = 2758^a^	16–25 *N* = 1 002 114^a^	26–35 *N* = 1 314 084^a^	36+ *N* = 142 554^a^	Overall *N* = 2 461 510^a^
Maternal characteristics	Maternal socioeconomic index during childhood	1 437 334					
	Medium		279 (16.5%)	131 571 (23.3%)	249 690 (31.2%)	22 473 (32.3%)	404 013 (28.1%)
	High		140 (8.3%)	51 542 (9.1%)	85 096 (10.6%)	8706 (12.5%)	145 484 (10.1%)
	Low		971 (57.5%)	304 474 (53.9%)	353 841 (44.2%)	26 529 (38.2%)	685 815 (47.7%)
	Agriculture		169 (10.0%)	56 866 (10.1%)	91 079 (11.4%)	9143 (13.2%)	157 257 (10.9%)
	Other		130 (7.7%)	20 459 (3.6%)	21 505 (2.7%)	2671 (3.8%)	44 765 (3.1%)
	(Missing)		1069	437 202	512 873	73 032	1 024 176
	Maternal immigration background	2 461 510					
	Domestic (two domestic parents)		2342 (84.9%)	887 850 (88.6%)	1 181 587 (89.9%)	129 371 (90.8%)	2 201 150 (89.4%)
	Foreign ancestry/foreign-born		416 (15.1%)	114 264 (11.4%)	132 497 (10.1%)	13 183 (9.2%)	260 360 (10.6%)
	Paternal age at birth (years)	2 461 510					
	<16		76 (2.8%)	119 (0.0%)	1 (0.0%)	0 (0.0%)	196 (0.0%)
	16–20		922 (33.4%)	30 191 (3.0%)	521 (0.0%)	13 (0.0%)	31 647 (1.3%)
	20+		576 (20.9%)	646 772 (64.5%)	948 809 (72.2%)	96 833 (67.9%)	1 692 990 (68.8%)
	Not defined		1184 (42.9%)	325 032 (32.4%)	364 753 (27.8%)	45 708 (32.1%)	736 677 (29.9%)
Offspring characteristics	Year of birth	2 461 510	1969 (1963, 1976)	1974 (1967, 1984)	1983 (1974, 1989)	1987 (1982, 1991)	1980 (1971, 1988)
	Men		1444 (52.4%)	514 083 (51.3%)	675 732 (51.4%)	72 881 (51.1%)	1 264 140 (51.4%)
	Birth weight (kg)	1 727 937	3.34 (0.56)	3.48 (0.53)	3.55 (0.54)	3.55 (0.59)	3.52 (0.54)
	(Missing)		1754	453 355	271 195	7269	733 573
	Gestational age (weeks)	1 727 065	39.04 (2.42)	39.56 (1.84)	39.55 (1.74)	39.30 (1.88)	39.53 (1.79)
	(Missing)		1774	454 266	271 131	7274	734 445

a Mean (standard deviation); number (%).

The primary outcomes were cardiometabolic measures (BMI, SBP, and T2D), and secondary outcomes included cognitive function, stress resilience, depression, and anxiety. The median age of cohort I was 18 years and that of cohort II ranged from 43 to 48 years. Tables S1–S3 present outcomes among offspring during late adolescence and adulthood. In cohort I, among offspring of pregnancies <16 years, 14.5%, men were overweight, and their mean (±SD) SBP, cognitive scores, and stress resilience scores were 128.9 ± 10.9, 5.1 ± 1.9, and 5.1 ± 1.7, respectively. In cohort II, the cumulative incidence of T2D, depression, and anxiety was 5.95%, 22.3%, and 25.3% among offspring of pregnancies <16 years, respectively. Compared with pregnancies between 16 and 25 years, offspring from pregnancies before 16 had a higher likelihood of being overweight, higher SBP, lower cognitive function, and lower stress resilience. They also had a higher incidence of T2D, depression, and anxiety.

**Figure 1:**
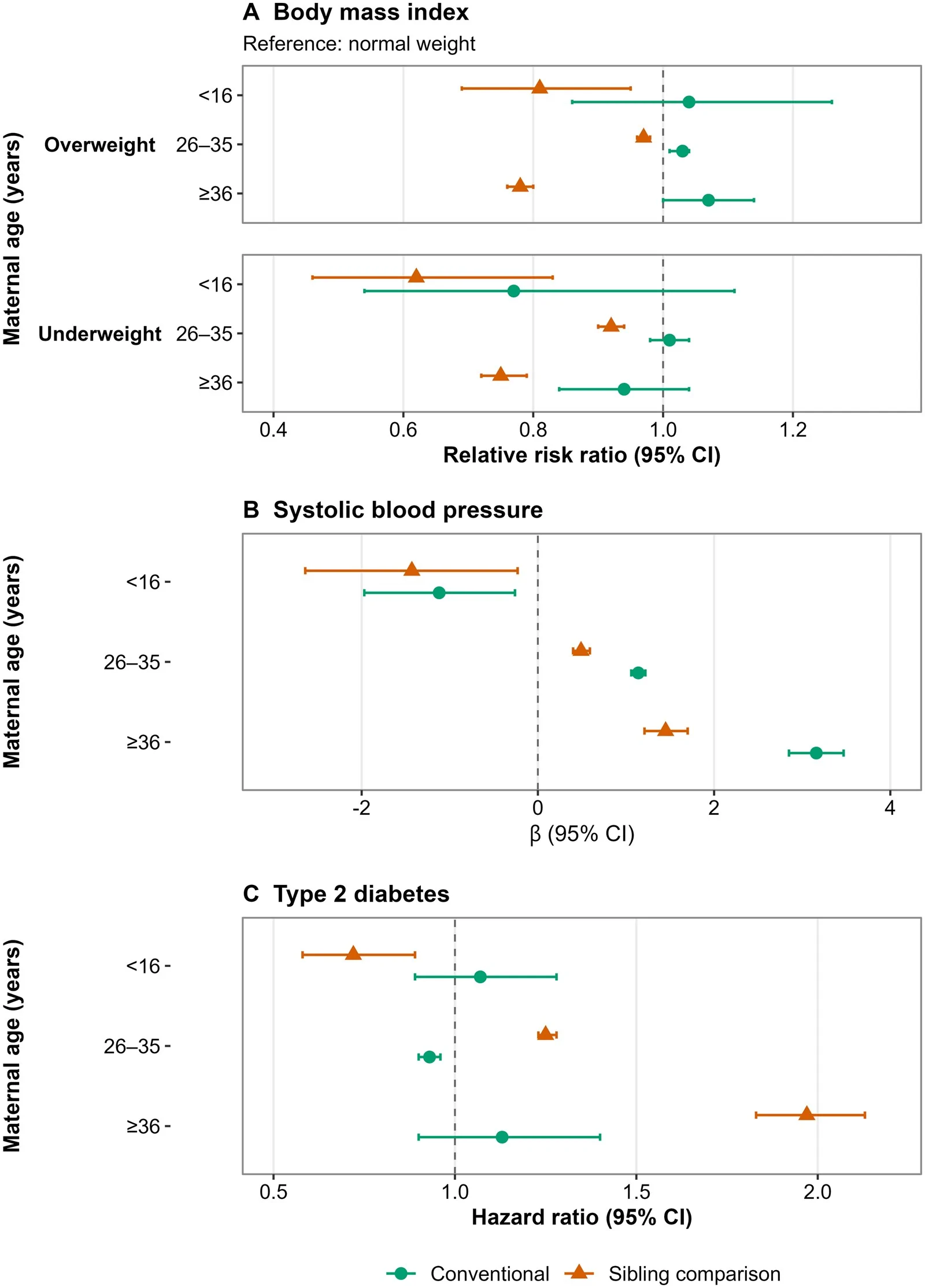
Associations of pregnancy age <16, 26–35, and ≥36 years (compared with 16–25 years) with late adolescent and adult offspring body mass index (BMI), systolic blood pressure, and type 2 diabetes in conventional and sibling-comparison analyses. Estimates are shown with 95% confidence intervals. Panel A presents relative risk ratios for overweight (BMI ≥25 kg/m²) and underweight (BMI <18.5 kg/m²), compared with normal weight (BMI 18.5–24.9 kg/m²). Panel B presents beta coefficients for systolic blood pressure. Panel C presents hazard ratios for type 2 diabetes. CI: confidence interval.

Figure 1 shows the associations between maternal age at delivery and offspring BMI, SBP, and T2D. In conventional analyses, maternal age <16 years was not associated with overweight or underweight but was associated with lower SBP. In sibling-comparison analyses, it was associated with lower risks of both overweight and underweight, with a stronger inverse association for SBP. In cohort II, conventional analyses showed little association with T2D, whereas sibling-comparison analyses indicated a lower risk.

Results for secondary outcomes are presented in Tables 3 and 4. In cohort I, in conventional analyses, maternal age <16 years was associated with poorer cognitive function and lower stress resilience in late adolescence (Table 3). These associations attenuated towards the null in sibling-comparison analyses. In cohort II, conventional analyses showed weak or inconsistent associations of maternal age <16 years with depression and anxiety (Table 4), whereas sibling-comparison analyses indicated lower risks. In sex-stratified analyses, conventional models showed lower anxiety in men and lower depression and anxiety in women. In sibling-comparison analyses, inverse associations with depression and anxiety were observed in both sexes.

**Table 3:** Cohort I—association of maternal age at delivery with health and developmental outcomes among 17–21 years old offspring.

Maternal age at birth (years)	Conventional^a^—M1 (unadjusted)		Conventional^a^—M2 (adjusted)		Sibling^b^—M1 (FE) (unadjusted)		Sibling^b^—M2 (FE) (adjusted)	
Cognitive function score								
<16	−0.60 (−0.71, −0.49)	< 1 × 10^−16^	−0.54 (−0.67, −0.40)	7.37 × 10^−15^	0.13 (−0.03, 0.29)	0.1499	0.13 (−0.05, 0.30)	0.1540
26–35	0.46 (0.45, 0.47)	< 1 × 10^−16^	0.45 (0.44, 0.46)	< 1 × 10^−16^	−0.26 (−0.27, −0.25)	< 1 × 10^−16^	−0.26 (−0.28, −0.25)	< 1 × 10^−16^
≥36	0.51 (0.49, 0.53)	< 1 × 10^−16^	0.53 (0.49, 0.57))	< 1 × 10^−16^	−0.46 (−0.48, −0.43)	< 1 × 10^−16^	−0.47 (−0.50, −0.44)	< 1 × 10^−16^
Resilience score								
<16	−0.34 (−0.44, −0.24)	1.32 × 10^−10^	−0.24 (−0.38, −0.10)	5.17 × 10^−4^	0.11 (−0.08, 0.29)	0.2571	0.11 (−0.08, 0.29)	0.2496
26–35	0.19 (0.19, 0.20)	< 1 × 10^−16^	0.21 (0.20, 0.22)	< 1 × 10^−16^	−0.26 (−0.27, −0.24)	< 1 × 10^−16^	−0.25 (−0.27, −0.24)	< 1 × 10^−16^
≥36	0.02 (−0.00, 0.04)	0.0502	0.32 (0.26, 0.34)	< 1 × 10^−16^	−0.49 (−0.52, −0.46)	< 1 × 10^−16^	−0.48 (−0.52, −0.45)	< 1 × 10^−16^

FE: fixed effect.

Reference maternal age category: 16–25.

a Conventional analysis.

Model 1 (M1): mother’s age at delivery.

Model 2 (M2): M1 + conscription age + maternal socioeconomic index during childhood (grandparental socioeconomic index) + maternal country of birth.

b Sibling-comparison analyses.

M1: mother’s age at delivery.

M2: M1 + conscription age.

**Table 4:** Cohort II—association of maternal age at delivery with health outcomes in adult offspring.

Outcome	Model	Maternal age (years)	Overall HR (95% CI), *P* value		Men HR (95% CI), *P* value		Women HR (95% CI), *P* value	
Type 2 diabetes			**M1—unadjusted**	**M2—adjusted**	**M1—unadjusted**	**M2—adjusted**	**M1—unadjusted**	**M2—adjusted**
	Conventional^a^	<16	1.20 (1.07–1.34), *P *= 1.88 × 10^−3^	1.07 (0.89–1.28), *P *= .47	1.28 (1.10–1.48), *P *= 1.13 × 10^−3^	1.14 (0.90–1.44), *P *= .30	1.10 (0.92–1.31), *P *= .30	0.99 (0.75–1.30), *P *= .10
		26–35	0.99 (0.98–1.00), *P *= 9.85 × 10^−5^	0.93 (0.90–0.96), *P *= 2.82 × 10^−5^	0.96 (0.94–0.98), *P *= 3.09 × 10^−9^	0.88 (0.85–0.92), *P *= 4.07 × 10^−10^	1.03 (1.01–1.04), *P *= 1.15 × 10^−2^	0.98 (0.94–1.02), *P *= .37
		36+	1.18 (1.12–1.23), *P *= 6.93 × 10^−3^	1.13 (0.90–1.40), *P *= .29	1.13 (1.06–1.20), *P *= 6.94 × 10^−12^	0.83 (0.58–1.17), *P *= .28	1.24 (1.16–1.32), *P *= 6.94 × 10^−12^	1.47 (1.11–1.95), *P *= 7.53 × 10^−3^
	Sibling^b^	<16	0.71 (0.58–0.88), *P *= 1.70 × 10^−3^	0.72 (0.58–0.89), *P *= 2.11 × 10^−3^	0.77 (0.54–1.11), *P *= .17		0.69 (0.44–1.08), *P *= .11	
		26–35	1.25 (1.22–1.28), *P *< 1 × 10^−16^	1.25 (1.23–1.28), *P *< 1 × 10^−16^	1.24 (1.19–1.29), *P *< 1 × 10^−16^		1.33 (1.27–1.39), *P *< 1 × 10^−16^	
		36+	1.97 (1.82–2.13), *P *< 1 × 10^−16^	1.97 (1.83–2.13), *P *< 1 × 10^−16^	1.82 (1.60–2.08), *P *< 1 × 10^−16^		2.48 (2.14–2.88), *P *< 1 × 10^−16^	
Depression	Conventional^a^	<16	1.08 (1.02–1.15), *P *= 1.18 × 10^−2^	0.94 (0.87–1.01), *P *= .11	1.13 (1.03–1.25), *P *= 1.07 × 10^−2^	1.02 (0.91–1.16), *P *= .72	1.06 (0.98–1.15), *P *= .17	0.88 (0.79–0.98), *P *= 1.94 × 10^−2^
		26–35	1.22 (1.22–1.23), *P *< 1 × 10^−16^	1.19 (1.18–1.20), *P *< 1 × 10^−16^	1.22 (1.21–1.23), *P *< 1 × 10^−16^	1.16 (1.15–1.17), *P *< 1 × 10^−16^	1.24 (1.23–1.24), *P *< 1 × 10^−16^	1.21 (1.20–1.22), *P *< 1 × 10^−16^
		36+	1.86 (1.84–1.89), *P *< 1 × 10^−16^	1.65 (1.62–1.68), *P *< 1 × 10^−16^	1.83 (1.80–1.87), *P *< 1 × 10^−16^	1.60 (1.56–1.65), *P *< 1 × 10^−16^	1.91 (1.88–1.94), *P *< 1 × 10^−16^	1.69 (1.65–1.72), *P *< 1 × 10^−16^
	Sibling^b^	<16	0.63 (0.57–0.70), *P *< 1 × 10^−16^	0.63 (0.57–0.70), *P *< 1 × 10^−16^	0.62 (0.50–0.77), *P *= 9.960e-06		0.59 (0.49–0.70), *P *= 8.90 × 10^−9^	
		26–35	1.82 (1.81–1.84), *P *< 1 × 10^−16^	1.85 (1.84–1.87), *P *< 1 × 10^−16^	1.82 (1.78–1.85), *P *< 1 × 10^−16^		2.03 (2.00–2.06), *P *< 1 × 10^−16^	
		36+	4.33 (4.23–4.42), *P *< 1 × 10^−16^	4.45 (4.35–4.55), *P *< 1 × 10^−16^	4.28 (4.09–4.48), *P *< 1 × 10^−16^		5.19 (5.00–5.40), *P *< 1 × 10^−16^	
Anxiety	Conventional^a^	<16	1.04 (0.97–1.11), *P *= .29	0.87 (0.80–0.95), *P *= 1.37 × 10^−3^	1.13 (1.02–1.25), *P *= 2.19 × 10^−2^	0.98 (0.86–1.11), *P *= .72	0.98 (0.90–1.08) *P *= .70	0.79 (0.71–0.89), *P *= 1.15 × 10^−4^
		26–35	1.29 (1.28–1.30), *P *< 1 × 10^−16^	1.26 (1.25–1.27), *P *< 1 × 10^−16^	1.27 (1.26–1.29), *P *< 1 × 10^−16^	1.23 (1.21–1.24), *P *< 1 × 10^−16^	1.31 (1.30–1.32), *P *< 1 × 10^−16^	1.29 (1.27–1.30), *P *< 1 × 10^−16^
		36+	2.04 (2.01–2.06), *P *< 1 × 10^−16^	1.82 (1.79–1.85), *P *< 1 × 10^−16^	1.99 (1.96–2.03), *P *< 1 × 10^−16^	1.74 (1.69–1.80), *P *< 1 × 10^−16^	2.09 (2.06–2.12), *P *< 1 × 10^−16^	1.87 (1.83–1.92), *P *< 1 × 10^−16^
	Sibling^b^	<16	0.54 (0.49–0.61), *P *< 1 × 10^−16^	0.55 (0.49–0.61), *P *< 1 × 10^−16^	0.56 (0.45–0.70), *P *= 7.71 × 10^−7^		0.43 (0.35–0.54), *P *= 6.91 × 10^−13^	
		26–35	1.98 (1.96–2.00), *P *< 1 × 10^−16^	2.01 (1.99–2.03), *P *< 1 × 10^−16^	2.00 (1.95–2.04), *P *< 1 × 10^−16^		2.20 (2.16–2.24), *P *< 1 × 10^−16^	
		36+	5.51 (5.38–5.65), *P *< 1 × 10^−16^	5.62 (5.49–5.76), *P *< 1 × 10^−16^	5.42 (5.16–5.69) *P *< 1 × 10^−16^		6.46 (6.19–6.74), *P *< 1 × 10^−16^	

HR: hazard ratio; CI: confidence interval.

Reference maternal age category: 16–25.

a Conventional analysis.

M1: mother’s age at delivery.

M2: M1 + maternal socioeconomic index during childhood (grandparental socioeconomic index) + maternal country of birth.

b Sibling-comparison analyses.

M1: mother’s age at delivery.

Sensitivity analyses showed results consistent with the main findings. Additional adjustment for paternal age, birth weight, and gestational age had minimal impact. Sibling-comparison estimates attenuated towards the null but remained directionally consistent (Tables S6–S8). Stratified analyses by grandparental SEI showed similar patterns across groups (Tables S9–S13). Protective associations, particularly for T2D, depression, and anxiety, were more pronounced among females and in sibling-comparison models (Tables S14–S21).

## Discussion

We examined the associations between pregnancies before age 16 years and offspring outcomes from late adolescence to adulthood using Swedish register data. In conventional analyses, offspring of mothers aged <16 years had lower SBP, poorer cognitive function, and lower stress resilience at ages 17–21. In sibling-comparison analyses, associations with underweight and overweight became inverse, and the inverse association with SBP strengthened. Associations with cognitive function and stress resilience attenuated to null. For adult outcomes, conventional analyses showed a lower risk of anxiety. In sibling-comparison analyses, associations with T2D and depression became inverse, and the inverse association with anxiety increased.

Sex-stratified analyses suggested stronger protective associations for depression and anxiety among females compared with males. The stronger protective associations observed among females may reflect differences in biological susceptibility, behavioural factors, or social pathways [39]. Although these mechanisms require further investigation, the findings were consistent in sensitivity analyses, including adjustment for paternal age, birth weight, and gestational age. Results were also similar across socioeconomic strata. Some variation by sex and birth cohort was observed, likely reflecting changes in social context. Overall, there was no evidence of adverse biological effects associated with very young maternal age.

Conscription assessment-based measures supported a life-course interpretation. Early cardiometabolic outcomes (BMI and SBP) showed patterns similar to adult outcomes in sibling-comparison analyses. This supports their role as early markers influenced by shared familial factors.

Aizer et al. [4] used a cousin-comparison design and reported poorer cognitive function among the oldest male offspring of mothers who had teenage pregnancies compared with cousins born to a maternal sister who became pregnant after adolescence. While informative, this design does not fully eliminate family-level confounding, as cousins may experience meaningfully different childhood environments and parental investments, and maternal characteristics can differ substantially between sisters [12]. In contrast, within-mother sibling designs control for all time-invariant maternal characteristics and shared familial environment, thereby strengthening internal validity [12, 13].

Myrskylä et al. [8] used sibling-comparison analyses in Swedish conscription assessment data and similarly reported poorer cognitive function among adult males born to mothers who experienced teenage pregnancies, while additionally conditioning on birth order. In contrast, we did not adjust for birth order. In our study, 99.9% of mothers with pregnancies before age 16 years had only one recorded pregnancy, resulting in a strong correlation between maternal age and birth order. Myrskylä et al. defined exposure using broader teenage pregnancy categories (15–19 years), within which birth order may vary more independently. Consequently, adjusting for birth order in our study would likely constitute overadjustment and obscure the total effect of maternal age [40]. However, given mixed evidence on birth order, some protective associations may partly reflect birth order effects [41–45].

Prior studies defined teenage pregnancies broadly (<19 years), whereas we focused on pregnancies before age 16. This corresponds to the peripubertal period, when insulin resistance peaks. This cut-off was hypothesis-driven as there is evidence that insulin resistance peaks at peripubertal ages (approximately 11–15 years), providing biological plausibility for possible long-term cardiometabolic consequences of peripubertal pregnancies [15, 17].

In contrast with our original hypothesis, that there may be long-term adverse metabolic or other health consequences for offspring born to peripubertal mothers, the results suggest that the in-utero environment and psychological immaturity of peripubertal-aged mothers do not usually lead to adverse late adolescent and adult outcomes in their offspring. Instead, the attenuation or reversal of associations with adverse outcomes in sibling-comparison models indicates that the elevated risks seen in conventional models are largely explained by unmeasured shared familial characteristics, such as material disadvantage and caregiving practices that have consequences for all offspring within the family.

These findings highlight that policies addressing teenage pregnancies should extend beyond prevention alone and incorporate broader social and economic interventions that support disadvantaged families such as social support, education, and income-related policies [46]. Overall, our results indicate that downstream support strategies may be as important as, or more relevant than, preventive efforts alone.

A key strength is the high representativeness of the conscription cohort, as only men with severe illness or disability were exempt. This likely bias estimates towards the null, yielding conservative effect estimates. Another strength is the use of data from national registers that covered the total population enabling study of outcomes in adult offspring. This provided near-complete follow-up, with loss to follow-up limited to emigration and death. The prospective nature of the data reduces biases inherent to self-reported data. The use of a sibling-comparison design strengthens causal inference by addressing unmeasured familial confounding [13]. Together, these features support high internal and external validity.

Potential weaknesses of the study include non-adjustment for maternal relationship status, which is not recorded for mothers aged <16 and could introduce differential bias. Women were excluded from the conscription assessment limiting generalizability. Multivariable analyses were conducted using a complete-case approach. This may introduce bias if the missingness is not completely at random. However, the direction of the associations was consistent, and the effect estimates were of similar magnitude in both the unadjusted and adjusted conventional analyses and sibling-comparison analyses.

While diagnoses recorded in registers have generally been demonstrated to have good positive predictive values [34, 47], some outcomes may be under-ascertained, particularly those managed in primary care or before outpatient data became available in 2001. T2D ascertainment may also miss milder cases treated without medication. Although we included diabetes cases at age 30 years or above, some misclassification may remain. Any misclassification mentioned above is likely to be non-differential and may attenuate associations. The large sample provided sufficient precision for the main analysis. However, the smaller number of exposed individuals within sibling-comparison models resulted in wider confidence intervals and should be interpreted cautiously.

Another limitation of sibling designs is that they do not adjust for unshared factors, which could introduce or even amplify residual confounding [48]. Moreover, sibling-comparison estimates reflect within-family effects in a subset of families and may not be fully generalizable to those without siblings or families without necessary variation in the family. Additionally, we lacked data on some maternal characteristics that vary between pregnancies.

## Conclusions

The findings of this study suggest that late-adolescent and adult offspring of mothers who were pregnant before age 16 years do not experience worse metabolic or developmental outcomes compared with their siblings born when the mothers were older. The adverse associations observed in conventional analyses appear largely attributable to shared familial characteristics rather than to the physiological or psychosocial immaturity associated with very young maternal age. Differences by birth order may contribute to some of the apparently advantageous outcomes observed among offspring born to younger mothers relative to their later-born siblings; however, these should be further investigated in future studies.

## Ethics approval

The study was approved by Swedish Ethical Review Authority (2019–04755 and 2023–05817-01).

## Supplementary Material

dyag153_Supplementary_Data

## Data Availability

The data used in this study cannot be shared publicly due to regulations under the relevant Swedish laws. Researchers who are interested in Swedish register data can refer to https://www.registerforskning.se/en/.

## References

[1] Pevalin DJ. Outcomes in Childhood and Adulthood by Mother’s Age at Birth: Evidence from the 1970 British Cohort Study. ISER Working Paper Series No. 2003–31. Colchester: University of Essex, 2003. https://www.iser.essex.ac.uk/research/publications/working-papers/iser/2003-31 (4 July 2026, date last accessed).

[2] Lipman EL , Georgiades K, Boyle MH. Young adult outcomes of children born to teen mothers: effects of being born during their teen or later years. J Am Acad Child Adolesc Psychiatry 2011;50:232–41.e4. 10.1016/j.jaac.2010.12.007 21334563

[3] Duncan GJ , Lee KTH, Rosales-Rueda M et al Maternal age and child development. Demography 2018;55:2229–55. 10.1007/s13524-018-0730-3 PMC6392079 30387046

[4] Aizer A , Devereux PJ, Salvanes KG. Grandparents, Moms, or Dads? Why Children of Teen Mothers Do Worse in Life. National Bureau of Economic Research Working Paper Series No. 25165. Cambridge, MA: National Bureau of Economic Research, 2018. https://www.nber.org/papers/w25165 (4 July 2026, date last accessed).

[5] Geronimus AT , Korenman S, Hillemeier MM. Does young maternal age adversely affect child development? Evidence from cousin comparisons in the United States. Popul Dev Rev 1994;20:585–609. 10.2307/2137602

[6] Francesconi M. Adult outcomes for children of teenage mothers. Scand J Econ 2008;110:93–117. 10.1111/j.1467-9442.2008.00526.x

[7] Myrskylä M , Fenelon A. Maternal age and offspring adult health: evidence from the health and retirement study. Demography 2012;49:1231–57. 10.1007/s13524-012-0132-x PMC3881604 22926440

[8] Myrskylä M , Silventoinen K, Tynelius P et al Is later better or worse? Association of advanced parental age with offspring cognitive ability among half a million young Swedish men. Am J Epidemiol 2013;177:649–55. 10.1093/aje/kws237 23467498

[9] Coyne CA , Långström N, Lichtenstein P et al The association between teenage motherhood and poor offspring outcomes: a national cohort study across 30 years. Twin Res Hum Genet 2013;16:679–89. 10.1017/thg.2013.23 PMC3657321 23632141

[10] Harden KP , Lynch SK, Turkheimer E et al A behavior genetic investigation of adolescent motherhood and offspring mental health problems. J Abnorm Psychol 2007;116:667–83. 10.1037/0021-843x.116.4.667 PMC2903734 18020715

[11] Imamura M , Tucker J, Hannaford P et al; REPROSTAT 2 group. Factors associated with teenage pregnancy in the European Union countries: a systematic review. Eur J Public Health 2007;17:630–6. 10.1093/eurpub/ckm014 17387106

[12] D’Onofrio BM , Lahey BB, Turkheimer E et al Critical need for family-based, quasi-experimental designs in integrating genetic and social science research. Am J Public Health 2013;103:S46–55. 10.2105/ajph.2013.301252 PMC3778076 23927516

[13] Sjölander A , Frisell T, Öberg S. Sibling comparison studies. Annu Rev Stat Appl 2022;9:71–94. 10.1146/annurev-statistics-040120-024521

[14] Emmanuel M , Bokor BR. Tanner stages. In: *StatPearls* [Internet]. Treasure Island (FL): StatPearls Publishing, 2025. https://www.ncbi.nlm.nih.gov/books/NBK470280/ (4 July 2026, date last accessed). 29262142

[15] Goran MI , Gower BA. Longitudinal study on pubertal insulin resistance. Diabetes 2001;50:2444–50. 10.2337/diabetes.50.11.2444 11679420

[16] Guarneri AM , Kamboj MK. Physiology of pubertal development in females. Pediatr Med 2019;2:42. 10.21037/pm.2019.07.03

[17] Moran A , Jacobs DR Jr, Steinberger J et al Insulin resistance during puberty: results from clamp studies in 357 children. Diabetes 1999;48:2039–44. 10.2337/diabetes.48.10.2039 10512371

[18] Ryan EA. Hormones and insulin resistance during pregnancy. Lancet 2003;362:1777–8. 10.1016/S0140-6736(03)14942-2 14654313

[19] Lacroix M , Kina E, Hivert MF. Maternal/fetal determinants of insulin resistance in women during pregnancy and in offspring over life. Curr Diab Rep 2013;13:238–44. 10.1007/s11892-012-0360-x 23307191

[20] Gluckman PD , Hanson MA, Pinal C. The developmental origins of adult disease. Matern Child Nutr 2005;1:130–41. 10.1111/j.1740-8709.2005.00020.x PMC6860944 16881892

[21] Reid V , Meadows-Oliver M. Postpartum depression in adolescent mothers: an integrative review of the literature. J Pediatr Health Care 2007;21:289–98. 10.1016/j.pedhc.2006.05.010 17825726

[22] Bergh C , Udumyan R, Fall K et al Stress resilience and physical fitness in adolescence and risk of coronary heart disease in middle age. Heart 2015;101:623–9. 10.1136/heartjnl-2014-306703 PMC4396533 25740818

[23] Bijwaard GE , van Kippersluis H, Veenman J. Education and health: the role of cognitive ability. J Health Econ 2015;42:29–43. 10.1016/j.jhealeco.2015.03.003 PMC4478201 25912224

[24] Crump C , Sundquist J, Winkleby MA et al Low stress resilience in late adolescence and risk of hypertension in adulthood. Heart 2016;102:541–7. 10.1136/heartjnl-2015-308597 PMC4794252 26830662

[25] Ji S , Chen Y, Zhou Y et al Association between anxiety and metabolic syndrome: an updated systematic review and meta-analysis. Front Psychiatry 2023;14:1118836. 10.3389/fpsyt.2023.1118836 PMC9978147 36873213

[26] Qiu W , Cai X, Zheng C et al Update on the relationship between depression and neuroendocrine metabolism. Front Neurosci 2021;15:728810. 10.3389/fnins.2021.728810 PMC8438205 34531719

[27] Batty GD , Wennerstad KM, Smith GD et al IQ in early adulthood and mortality by middle age: cohort study of 1 million Swedish men. Epidemiology 2009;20:100–9. 10.1097/EDE.0b013e31818ba076 19234402

[28] Axelsson O. The Swedish medical birth register. Acta Obstet Gynecol Scand 2003;82:491–2. 10.1034/j.1600-0412.2003.00172.x 12780418

[29] Ekbom A. The Swedish multi-generation register. Methods Mol Biol 2011;675:215–20. 10.1007/978-1-59745-423-0_10 20949391

[30] Ludvigsson JF , Almqvist C, Bonamy AK et al Registers of the Swedish total population and their use in medical research. Eur J Epidemiol 2016;31:125–36. 10.1007/s10654-016-0117-y 26769609

[31] Statistics Sweden. *Register-based Population and Housing Census*. https://www.scb.se/en/finding-statistics/statistics-by-subject-area/population-and-living-conditions/population-composition-and-development/register-based-population-and-housing-census (4 July 2026, date last accessed).

[32] Ludvigsson JF , Svedberg P, Olén O et al The longitudinal integrated database for health insurance and labour market studies (LISA) and its use in medical research. Eur J Epidemiol 2019;34:423–37. 10.1007/s10654-019-00511-8 PMC6451717 30929112

[33] Ludvigsson JF , Berglind D, Sundquist K et al The Swedish military conscription register: opportunities for its use in medical research. Eur J Epidemiol 2022;37:767–77. 10.1007/s10654-022-00887-0 PMC9329412 35810240

[34] Ludvigsson JF , Andersson E, Ekbom A et al External review and validation of the Swedish national inpatient register. BMC Public Health 2011;11:450. 10.1186/1471-2458-11-450 PMC3142234 21658213

[35] Brooke HL , Talbäck M, Hörnblad J et al The Swedish cause of death register. Eur J Epidemiol 2017;32:765–73. 10.1007/s10654-017-0316-1 PMC5662659 28983736

[36] Wettermark B , Hammar N, Fored CM et al The new Swedish Prescribed Drug Register—opportunities for pharmacoepidemiological research and experience from the first six months. Pharmacoepidemiol Drug Saf 2007;16:726–35. 10.1002/pds.1294 16897791

[37] Petersen AH , Lange T. What is the causal interpretation of sibling comparison designs? Epidemiology 2020;31:75–81. 10.1097/ede.0000000000001108 31651661

[38] Sjölander A , Öberg S, Frisell T. Generalizability and effect measure modification in sibling comparison studies. Eur J Epidemiol 2022;37:461–76. 10.1007/s10654-022-00844-x PMC9209381 35312926

[39] Mauvais-Jarvis F. Sex differences in metabolic homeostasis, diabetes, and obesity. Biol Sex Differ 2015;6:14. 10.1186/s13293-015-0033-y PMC4559072 26339468

[40] Pearl J. Causality: Models, Reasoning, and Inference. 2nd edn. Cambridge: Cambridge University Press, 2009. https://archive.illc.uva.nl/cil/uploaded_files/inlineitem/Pearl_2009_Causality (4 July 2026, date last accessed).

[41] Black SE , Devereux PJ, Salvanes KG. Healthy(?), wealthy, and wise: birth order and adult health. Econ Hum Biol 2016;23:27–45. 10.1016/j.ehb.2016.06.005 27442721

[42] Derraik JG , Ahlsson F, Lundgren M et al First-borns have greater BMI and are more likely to be overweight or obese: a study of sibling pairs among 26,812 Swedish women. J Epidemiol Community Health 2016;70:78–81. 10.1136/jech-2014-205368 26311896

[43] Jelenkovic A , Silventoinen K, Tynelius P et al Association of birth order with cardiovascular disease risk factors in young adulthood: a study of one million Swedish men. PLoS One 2013;8:e63361. 10.1371/journal.pone.0063361 PMC3656047 23696817

[44] Montgomery S , Bergh C, Udumyan R et al Sex of older siblings and psychological functioning. LLCS 2018;9:447–55. 10.14301/llcs.v9i4.486

[45] Paffenbarger RS Jr , Thorne MC, Wing AL. Chronic disease in former college students. VIII. Characteristics in youth predisposing to hypertension in later years. Am J Epidemiol 1968;88:25–32. 10.1093/oxfordjournals.aje.a120864 12418454

[46] Marmot M , Bell R. Fair society, healthy lives. Public Health 2012;126:S4–10. 10.1016/j.puhe.2012.05.014 22784581

[47] Everhov ÅH , Frisell T, Osooli M et al Diagnostic accuracy in the Swedish national patient register: a review including diagnoses in the outpatient register. Eur J Epidemiol 2025;40:359–69. 10.1007/s10654-025-01221-0 PMC12137447 40140143

[48] Frisell T. Invited commentary: sibling-comparison designs, are they worth the effort? Am J Epidemiol 2021;190:738–41. 10.1093/aje/kwaa183 PMC8096426 32830847

