# Supplementary material for: Peripubertal pregnancies and associations with health and developmental outcomes in offspring during late adolescence and adulthood: sibling-comparison register study
Source: Int J Epidemiol. 2026 Aug 27;55(5):dyag153. doi: 10.1093/ije/dyag153 (PMC13520932; doi:10.1093/ije/dyag153)
Supplement: Supplementary file 1 [file dyag153_supplementary_data.pdf]

Peripubertal pregnancies and associations with health and developmental outcomes in offspring during late adolescence and adulthood: sibling-comparison register study

Supplementary material

Table of Contents

Supplementary methods 4

Study sample 4

Outcomes 4

Cohort I 4

Cohort II 4

Covariates 4

Statistical analysis 5

Sensitivity analyses 5

Supplementary tables 6

Table S1. Cohort I- based on conscription participation- distribution of body mass index among 17-21 years old offspring by maternal age at pregnancy 6

Table S2. Cohort I: distribution of other health and development outcomes among 17-21 years old offspring by maternal age at pregnancy 6

Table S3. Cohort II - based on complete Swedish register data - incidence of type 2 diabetes, anxiety, and depression in adult offspring 7

Table S4. Cohort I: family counts and maternal-age discordance in sibling-comparison analyses 8

Table S5. Cohort II: family counts and maternal-age discordance in sibling-comparison analyses 8

Table S6: Cohort I: association of maternal age at delivery with body mass index among 17-21 years old offspring at conscription–assessment in sensitivity analyses 9

Table S7: Cohort I: association of maternal age at delivery with health and development among 17-21 years old offspring at conscription–assessment in sensitivity analyses 11

Table S8: Cohort II - association of maternal age at delivery with health outcomes in adult offspring in sensitivity analyses 13

Table S9. Cohort I - association of maternal age at delivery and body mass index of offspring at conscription-assessment – stratification by grandparental socioeconomic index 15

Table S10. Cohort I - association of maternal age at delivery and health and development outcomes at conscription–assessment stratification by grandparental socioeconomic index 16

Table S11. Cohort II - association of maternal age at delivery and type 2 diabetes in adult offspring – stratified by grandparental socioeconomic index 17

Table S12. Cohort II - association of maternal age at delivery and adult offspring depression – stratified by grandparental socioeconomic index 18

Table S13. Cohort II - association of maternal age at delivery and adult offspring anxiety – stratified by grandparental socioeconomic index 18

Table S14. Cohort I - association of maternal age at delivery and body mass index of offspring at conscription – effect modification by birth cohort 19

Table S15. Cohort I - association of maternal age at delivery and body mass index of offspring at conscription – effect modification by birth cohort ... 20
Table S16. Cohort II - association of maternal age at delivery and type 2 diabetes in adult offspring– effect modification by birth cohort ... 21
Table S17. Cohort II - association of maternal age at delivery and depression in adult offspring– effect modification by birth cohort ... 21
Table S18. Cohort II - association of maternal age at delivery and anxiety in adult offspring– effect modification by birth cohort ... 22
Table S19. Cohort II - association of maternal age at delivery and type 2 diabetes in adult offspring– effect modification by sex ... 22
Table S20. Cohort II - association of maternal age at delivery and depression in adult offspring– effect modification by sex ... 23
Table S21. Cohort II - association of maternal age at delivery and anxiety in adult offspring– effect modification by sex ... 23
References ... 24

Supplementary methods

Study sample

Cohort I participants were born between 1951 and 1988 and underwent mandatory military conscription examinations. Around 90% of men generally participated in conscription during this time [1]. Men were excluded if information on maternal age at delivery was missing.

Cohort II participants were born between 1946 and 1994 and were followed through national registers during the study period. Cohort members were excluded if maternal age at delivery was unavailable. The sibling-comparison sub-cohort consisted of 2,151,783 offspring born to 875,368 mothers.

Outcomes

Cohort I

Information on outcomes among late adolescent and young adult males was obtained from the MCR. Body mass index (BMI) was calculated as weight in kilograms divided by height in meters squared, BMI=weight(kg)/height2(m^2) [1]. Systolic blood pressure (SBP) in mmHg was measured as published previously [1]. Cognitive function and stress resilience were measured as part of the conscription assessment and graded on normally distributed 9-level scales, as published previously [1-3]. Cognitive function measured conscripts' range of mental abilities that are essential for everyday functioning such as thinking, learning, memorising, and making decisions [2,3]. Stress resilience assessed the potential conscripts' ability to cope with stress based on responses to everyday life situations [4]. Lower resilience to stress implies more frequent and prolonged stressful experiences.

Cohort II

Information on adult offspring outcomes was obtained using the NPR, NCDR, and NPDR. Diagnoses in these registers are coded using the Swedish version of International Classification of Disease (ICD) [5]. For this study we extracted the information between for the years 1990 to 2018 using ICD-9 (1987-1996) and ICD-10 (1997-). ICD codes 250 (ICD-9) and E11 & E14 (ICD-10) were used to identify type 2 diabetes (T2D). ICD codes 296, 298, and 311 (ICD-9) and F32-33 (ICD-10) were used for depression diagnoses. ICD codes 300 (ICD-9) and F41 (ICD-10) were used for anxiety diagnoses. The NPDR records dispensed prescriptions since 2005, coded using the Anatomical Therapeutic Chemical (ATC) Classification system [6]. ATC group A10 (insulin and oral glucose-lowering drugs), N06A, and N05B were used to identify T2D, depression and anxiety, respectively [6]. ICD-9 and the use of insulin and glucose lowering drugs to identify diabetes mellitus does not differentiate between types of diabetes. Thus, to approximately differentiate T2D from type 1 diabetes and other forms of diabetes, age of onset of diabetes at age ≥30 years was used. The first available record of these diagnoses between 1990 and 2018 were used as the incident occurrence.

Covariates

As sibling-comparison models control for all shared familial characteristics by design, persistent socioeconomic conditions and caregiving practices represented by grandparental SEI and maternal country of birth cannot be included in sibling-comparison analyses. Grandparental SEI was derived based on occupation and grouped into five categories (high, low, agriculture, middle and others) [7]. Maternal SEI at delivery was not used because for mothers <16 years, it reflects their parents' occupation rather than their own, thus using this would introduce differential misclassification between exposure groups. Maternal country of birth was defined as foreign-born, domestic born with two foreign-born parents, domestic

born with a domestic and a foreign-born parent, grouped into foreign ancestry/foreign-born and domestic born with two domestically born parents. In addition, foreign-born siblings were excluded as the conditions surrounding prior to immigration may have differed, potentially influencing long-term health.

Statistical analysis

Sensitivity analyses

Both in conventional and sibling-comparison analyses, models investigating associations of pregnancy <16 years compared with pregnancy between 16-25 years with late adolescent and adult offspring outcomes were additionally adjusted for paternal age to reduce confounding related to partner selection [8]. Subsequently, these were adjusted for birth weight and gestational age to evaluate the robustness of findings rather than as primary confounder control [9]. We stratified these associations by grandparental SEI grouped into three categories namely high, middle, and low/others (with agriculture, low, and others combined) [10]. We also tested whether these associations differed by sex (T2D, depression, and anxiety) [11]. Additionally, to address potential temporal changes in the association due to shifting social context and declining rates of pregnancy at a very young age, we assessed effect modification by offspring birth cohort, categorised into early vs late birth cohorts based on year of birth [12]. All analyses were performed in R version 4.5.1.

Supplementary tables

Table S1. Cohort I- based on conscription participation- distribution of body mass index among 17-21 years old offspring by maternal age at pregnancy

Maternal age at pregnancy (years)	Normal weight n (%)	Underweight n (%)	Overweight n (%)
Overall	645907 (79.0%)	53836 (6.6%)	118214 (14.5%)
<16	857 (77.1%)	54 (4.9%)	201 (18.1%)
16-25	295684 (79.0%)	25257 (6.8%)	53201 (14.2%)
26-35	324122 (79.1%)	26411 (6.4%)	59140 (14.4%)
36+	25244 (76.4%)	2114 (6.4%)	5672 (17.2%)

Table S2. Cohort I: distribution of other health and development outcomes among 17-21 years old offspring by maternal age at pregnancy

Maternal age at pregnancy (years)	Number	Systolic blood pressure (mmHg) mean (SD)	Cognitive function scores, mean (SD)	Resilience scores mean (SD)
Overall	943,444	128.9 (10.9)	5.1 (1.9)	5.1 (1.7)
<16	1,226	127.8 (11.0)	4.3 (1.7)	4.6 (1.7)
16-25	418,341	128.4 (10.8)	4.9 (1.9)	5.0 (1.7)
26-35	481,479	129.2 (10.9)	5.3 (1.9)	5.1 (1.7)
36+	42,398	129.9 (11.1)	5.4 (1.9)	5.0 (1.8)

SD, standard deviation

Table S3. Cohort II - based on complete Swedish register data - incidence of type 2 diabetes, anxiety, and depression in adult offspring

Outcome	Maternal age at pregnancy (years)	N at risk	Events (n)	Incidence (%)	Median follow-up, years (IQR)	Median age at diagnosis, years (IQR)
**Type 2 diabetes**	Overall	1,760,750	103,383	5.9	12.6 (5.4–19.0)	42.1 (36.0–48.7)
	<16	2,463	301	12.2	20.0 (12.6–24.7)	47.6 (40.1–52.9)
	16-25	809,887	63,021	7.8	16.5 (8.3–22.3)	44.4 (38.1–50.7)
	26-35	873,710	38,191	4.4	10.1 (4.3–16.1)	39.1 (34.3–44.8)
	36+	74,690	1,870	2.5	5.3 (2.4–9.3)	34.5 (32.0–38.5)
**Depression**	Overall	2,407,475	608,155	25.3	22.0 (10.4–26.9)	31.7 (25.0–39.7)
	<16	2,725	995	36.5	33.1 (18.2–34.6)	39.3 (32.0–45.7)
	16-25	985,921	275,075	27.9	27.9 (13.0–31.5)	35.8 (27.6–43.1)
	26-35	1,281,341	299,632	23.4	18.7 (9.5–23.7)	29.5 (24.1–36.8)
	36+	137,488	32,453	23.6	13.5 (7.9–16.6)	25.6 (22.3–30.3)
**Anxiety**	Overall	2,411,934	538,381	22.3	21.9 (10.5–27.5)	31.9 (24.9–40.4)
	<16	2,727	842	30.9	33.0 (19.5–35.6)	40.2 (32.5–47.0)
	16-25	987,572	238,502	24.2	27.8 (13.6–32.0)	36.4 (27.6–44.2)
	26-35	1,283,690	268,937	21.0	18.7 (9.6–24.2)	29.6 (24.0–37.2)
	36+	137,945	30,100	21.8	13.6 (8.0–16.8)	25.8 (22.3–30.5)

IQR, interquartile range

Table S4. Cohort I: family counts and maternal-age discordance in sibling-comparison analyses

Dataset	Families in sibling model	Families with ≥2 offspring	Exposure-discordant families [a]	Children in discordant families	Median offspring per family	IQR offspring per family
Body mass index	601,577	183,171	95,575	218,512	2	2–2
Systolic blood pressure, cognitive function, stress resilience	676,267	223,015	117,360	271,259	2	2–2

IQR, interquartile range

[a] Exposure-discordant families are those in which siblings differ in maternal age-at-birth category. Only exposure-discordant families contribute identifying information for maternal age effects in sibling comparison models.

Table S5. Cohort II: family counts and maternal-age discordance in sibling-comparison analyses

Outcome	Families in sibling model	Families with ≥2 offspring	Exposure-discordant families [a]	Event-discordant families [b]	Both exposure & event discordant	Median offspring per family	IQR offspring per family
Type 2 diabetes	905,782	609,228	332,070	78,605	49,309	2	1–2
Anxiety	1,167,017	855,147	463,855	320,316	194,738	2	1–3
Depression	1,166,606	853,223	462,731	342,226	208,637	2	1–3

IQR, interquartile range

[a] Exposure-discordant, siblings differ in maternal age at birth category; [b] event-discordant, siblings differ in the outcome (type 2 diabetes, anxiety, depression); both discordances are required for full identifiability of exposure effects.

Table S6: Cohort I: association of maternal age at delivery with body mass index among 17-21 years old offspring at conscription–assessment in sensitivity analyses

Outcome	Maternal age (years)	Underweight RRR (95% CI)				Overweight RRR (95% CI)			
		Conventional model		Sibling-comparison model		Conventional model		Sibling-comparison model	
**M1[a] - Paternal age at the time of birth**		**RRR (95% CI)**	**p value**	**RRR (95% CI)**	**p value**	**RRR (95% CI)**	**p value**	**RRR (95% CI)**	***p* value**
	**<16**	0.75 (0.52, 1.08)	0.12	0.86 (0.65, 1.13)	0.27	1.05 (0.86, 1.27)	0.66	0.79 (0.46, 1.37)	0.34
	**26-35**	1.01 (0.98, 1.04)	0.35	1.13 (1.11, 1.14)	$< 1 \times 10^{-16}$	1.02 (1.01, 1.04)	0.01	1.14 (1.11, 1.18)	$< 1 \times 10^{-16}$
	**36+**	0.94 (0.84, 1.04)	0.24	1.03 (1.00, 1.06)	0.08	1.07 (1.00, 1.14)	0.04	1.11 (1.05, 1.18)	4.11×10^{-3}
**M2[b] – birth weight & gestational age**									
	**<16**	0.87 (0.87, 0.87)	$< 1 \times 10^{-16}$	0.85 (0.64, 1.11)	0.23	1.13 (1.13, 1.13)	$< 1 \times 10^{-16}$	0.75 (0.43, 1.31)	0.32
	**26-35**	1.08 (1.08, 1.09)	$< 1 \times 10^{-16}$	1.15 (1.13, 1.17)	$< 1 \times 10^{-16}$	0.97 (0.95, 0.98)	0.0001.64	1.23 (1.19, 1.26)	$< 1 \times 10^{-16}$
	**36+**	1.02 (1.02, 1.02)	$< 1 \times 10^{-16}$	1.08 (1.04, 1.11)	1.21×10^{-5}	1.00 (1.00, 1.00)	1.11×10^{-14}	1.25 (1.18, 1.32)	1.59×10^{-13}

RRR, relative risk ratio; CI, confidence interval

Reference maternal age category: 16–25 years

[a] **M1: Model 1**

Conventional analyses: adjusted for mother's age at delivery, conscription age, grandparental socioeconomic index, maternal country of birth, and paternal age at the time of birth of the offspring

Sibling-comparison analyses. adjusted for mother's age at delivery, conscription age, and paternal age at the time of birth

[b] **M2: model 2**

Conventional analyses: adjusted for mother's age at delivery, conscription age, grandparental socioeconomic index, maternal country of birth, paternal age at the time of birth of the offspring, birth weight, and gestational age of the offspring

Sibling-comparison analyses. adjusted for mother's age at delivery, conscription age, paternal age at the time of birth of the offspring, birth weight, and gestational age of the offspring

Table S7: Cohort I: association of maternal age at delivery with health and development among 17-21 years old offspring at conscription–assessment in sensitivity analyses

Outcome	Maternal age (years)	Systolic blood pressure β (95% CI), *p* value		Cognitive function scores β (95% CI), *p* value		Resilience scores β (95% CI), *p* value	
**M1 [a] - Paternal age at the time of birth**		**Conventional model**	**Sibling-comparison model**	**Conventional model**	**Sibling-comparison model**	**Conventional model**	**Sibling-comparison model**
	**<16**	-0.75 (-1.61, 0.12), 8.97×10^{-2}	-2.42 (-5.94, 1.09), 0.18	-0.41 (-0.54, -0.27), 8.05×10^{-9}	0.22 (-0.21, 0.65), 0.32	-0.14 (-0.28, -0.01), 4.19×10^{-2}	-0.32 (-0.73, 0.10), 0.13
	**26-35**	1.11 (1.02, 1.19), $<1 \times 10^{-16}$	0.73 (0.54, 0.91), 8.29×10^{-15}	0.44 (0.43, 0.45), $<1 \times 10^{-16}$	-0.23 (-0.25, -0.21), $<1 \times 10^{-16}$	0.20 (0.18, 0.21), $<1 \times 10^{-16}$	-0.28 (-0.30, -0.26), $<1 \times 10^{-16}$
	**36+**	3.13 (2.82, 3.44), $<1 \times 10^{-16}$	1.95 (1.55, 2.35), $<1 \times 10^{-16}$	0.52 (0.48, 0.56), $<1 \times 10^{-16}$	-0.49 (-0.54, -0.45), $<1 \times 10^{-16}$	0.30 (0.26, 0.34), $<1 \times 10^{-16}$	-0.52 (-0.57, -0.47), $<1 \times 10^{-16}$
**M2 [b] – birth weight & gestational age**							
	**<16**	-1.20 (-2.54, 0.15), 8.18×10^{-2}	-2.46 (-5.97, 1.05), 0.17	-0.47 (-0.68, -0.27), 5.60×10^{-6}	0.23 (-0.20, 0.66), 0.30	-0.29 (-0.50, -0.07), 8.07×10^{-3}	-0.31 (-0.73, 0.10), 0.14
	**26-35**	0.99 (0.90, 1.07), $<1 \times 10^{-16}$	0.74 (0.55, 0.92), 1.27×10^{-14}	0.41 (0.40, 0.42), $< <1 \times 10^{-16}$	-0.24 (-0.26, -0.22), $<1 \times 10^{-16}$	0.19 (0.18, 0.20), $< <1 \times 10^{-16}$	-0.28 (-0.31, -0.26), $<1 \times 10^{-16}$
	**36+**	2.99 (2.67, 3.30), $<1 \times 10^{-16}$	1.93 (1.52, 2.33), $< <1 \times 10^{-16}$	0.49 (0.45, 0.53), $< <1 \times 10^{-16}$	-0.51 (-0.56, -0.47), $<1 \times 10^{-16}$	0.28 (0.24, 0.32), $< <1 \times 10^{-16}$	-0.53 (-0.58, -0.48), $<1 \times 10^{-16}$

CI, confidence interval

Reference maternal age category: 16–25

[a] **M1: model 1**

Conventional analyses: adjusted for mother's age at delivery, conscription age, grandparental socioeconomic index, maternal country of birth, and paternal age at the time of birth of the offspring

Sibling-comparison analyses. adjusted for mother's age at delivery, conscription age, and paternal age at the time of birth

[b] **M2: model 2**

Conventional analyses: adjusted for mother's age at delivery, conscription age, grandparental socioeconomic index, maternal country of birth, paternal age at the time of birth of the offspring, birth weight, and gestational age of the offspring

Sibling-comparison analyses. adjusted for mother's age at delivery, conscription age, paternal age at the time of birth of the offspring, birth weight, and gestational age of the offspring

Table S8: Cohort II - association of maternal age at delivery with health outcomes in adult offspring in sensitivity analyses

Outcome	Maternal age (years)	Type 2 diabetes HR (95% CI), *p* value		Depression HR (95% CI), *p* value		Anxiety HR (95% CI), *p* value	
**M1 [a] - Paternal age at the time of birth**		**Conventional model**	**Sibling-comparison model**	**Conventional model**	**Sibling-comparison model**	**Conventional model**	**Sibling-comparison model**
	**<16**	1.04 (0.87–1.25), 0.66	0.75 (0.60–0.92), 6.87×10^{-3}	0.93 (0.86–1.01), 5.12×10^{-2}	0.69 (0.62–0.77), 3.32×10^{-12}	0.86 (0.79–0.94), 8.00×10^{-4}	0.62 (0.55–0.69), 1.68×10^{-16}
	**26-35**	0.93 (0.90–0.96), 3.92×10^{-3}	1.25 (1.22–1.28), $<1 \times 10^{-16}$	1.18 (1.17–1.19), $<1 \times 10^{-16}$	1.84 (1.82–1.86), $<1 \times 10^{-16}$	1.26 (1.25–1.27), $<1 \times 10^{-16}$	1.99 (1.97–2.01), $<1 \times 10^{-16}$
	**36+**	1.12 (0.90–1.40), 0.31	1.97 (1.82–2.12), $<1 \times 10^{-16}$	1.63 (1.60–1.66), $<1 \times 10^{-16}$	4.40 (4.30–4.50), $<1 \times 10^{-16}$	1.82 (1.79–1.86), $<1 \times 10^{-16}$	5.56 (5.42–5.69), $<1 \times 10^{-16}$
**M2 [b] – birth weight & gestational age**							
	**<16**	1.49 (1.12–1.97), 5.53×10^{-3}	0.67 (0.33–1.36), 0.28	1.08 (0.97–1.21), 3.32×10^{-12}	0.65 (0.54–0.79), 1.83×10^{-16}	1.02 (0.91–1.15), 1.68×10^{-16}	0.62 (0.50–0.76), 4.24×10^{-4}
	**26-35**	0.91 (0.89–0.94), 3.32×10^{-9}	1.12 (1.07–1.18), 8.00×10^{-6}	1.10 (1.09–1.11), $<1 \times 10^{-16}$	1.70 (1.68–1.72), $<1 \times 10^{-16}$	1.16 (1.16–1.17), $< 1 \times 10^{-16}$	1.79 (1.77–1.82), $<1 \times 10^{-16}$
	**36+**	1.09 (0.87–1.35), 0.47	1.18 (1.03–1.34), 1.77×10^{-2}	1.50 (1.47–1.52), $<1 \times 10^{-16}$	3.31 (3.22–3.40), $<1 \times 10^{-16}$	1.66 (1.63–1.69), $< 1 \times 10^{-16}$	3.96 (3.84–4.08), $<1 \times 10^{-16}$

HR, hazard ratio; CI, confidence interval

Reference maternal age category: 16–25 years

[a] **M1: model 1**

Conventional analyses: adjusted for mother's age at delivery, sex, grandparental socioeconomic index, maternal country of birth, and paternal age at the time of birth

Sibling-comparison analyses. adjusted for mother's age at delivery, sex, and paternal age at the time of birth

[b] **M2: model 2**

Conventional analyses: adjusted for mother's age at delivery, sex, grandparental socioeconomic index, maternal country of birth, paternal age at the time of birth, birth weight, and gestational age of the offspring

Sibling-comparison analyses. adjusted for mother's age at delivery, sex, and paternal age at the time of birth, birth weight, and gestational age of the offspring

Table S9. Cohort I - association of maternal age at delivery and body mass index of offspring at conscription-assessment – stratification by grandparental socioeconomic index

Grandparental socioeconomic index	Maternal age at delivery (years)	Conventional model RRR (95% CI)	*p* value	Sibling-comparison model RRR (95% CI)	*p* value
**Underweight**					
**Medium**	**<16**	0.91 (0.37, 2.25)	0.83	0.69 (0.43, 1.11)	0.13
	**26–35**	1.07 (1.01, 1.13)	2.55×10^{-2}	1.02 (0.98, 1.06)	0.33
	**≥36**	1.03 (0.86, 1.23)	0.78	0.87 (0.78, 0.97)	1.33×10^{-2}
**High**	**<16**	0.90 (0.21, 3.75)	0.88	0.62 (0.31, 1.23)	0.17
	**26–35**	1.12 (1.02, 1.22)	0.20	1.04 (0.98, 1.10)	0.17
	**≥36**	1.06 (0.80, 1.41)	0.67	1.05 (0.89, 1.25)	0.55
**Low/Other**	**<16**	0.74 (0.49, 1.11)	0.15	1.10 (0.88, 1.38)	0.40
	**26–35**	0.97 (0.94, 1.01)	0.12	0.93 (0.91, 0.95)	7.56×10^{-11}
	**≥36**	0.86 (0.74, 1.00)	5.43×10^{-2}	0.92 (0.85, 1.00)	3.78×10^{-2}
**Overweight**					
**Medium**	**<16**	1.52 (0.96, 2.41)	7.34×10^{-2}	0.67 (0.27, 1.65)	0.39
	**26–35**	0.96 (0.93, 1.00)	3.15	1.07 (1.01, 1.14)	0.29×10^{-2}
	**≥36**	1.11 (0.99, 1.24)	6.18×10^{-2}	0.88 (0.72, 1.07)	0.20
**High**	**<16**	1.64 (0.85, 3.18)	0.14	0.61 (0.15, 2.42)	0.49
	**26–35**	0.95 (0.89, 1.00)	5.14×10^{-2}	1.15 (1.04, 1.27)	7.53×10^{-3}
	**≥36**	0.92 (0.77, 1.10)	0.37	1.13 (0.83, 1.54)	0.44
**Low/Other**	**<16**	0.93 (0.74, 1.16)	0.51	0.81 (0.52, 1.27)	0.36
	**26–35**	1.06 (1.03, 1.08)	4.70	0.90 (0.86, 0.93)	7.18×10^{-8}
	**≥36**	1.06 (0.97, 1.15)	0.19	0.78 (0.66, 0.92)	2.83×10^{-3}

RRR, relative risk ratio; CI, confidence interval

Reference maternal age category, 16–25 years**;** BMI reference category, normal weight

RRR shown for Underweight vs Normal and Overweight/Obese vs Normal within each maternal SEI category (Medium, High, Low/Other).

P-interaction are 1.13×10^{-7} for both underweight and overweight in conventional analyses from likelihood ratio test of maternal age × grandparental socioeconomic index interaction in the population multinomial model.

Table S10. Cohort I - association of maternal age at delivery and health and development outcomes at conscription–assessment stratification by grandparental socioeconomic index

Grandparental socioeconomic index	Maternal age at delivery	Conventional model β (95% CI)	*p* value	Sibling-comparison model β (95% CI)	*p* value
**Systolic blood pressure**					
**Medium**	<16	-1.30 (-3.48, 0.89)	0.35	-0.35 (-5.15, 4.45)	0.86
	26–35	0.99 (0.83, 1.15)	$<1 \times 10^{-16}$	0.58 (0.21, 0.94)	2.03×10^{-8}
	≥36	2.94 (2.39, 3.48)	$<1 \times 10^{-16}$	2.15 (1.10, 3.20)	5.56×10^{-6}
**High**	<16	-2.29 (-5.57, 0.99)	0.17	-1.74 (-8.11, 4.64)	0.59
	26–35	1.04 (0.79, 1.30)	3.49×10^{-16}	0.38 (-0.18, 0.94)	0.18
	≥36	2.64 (1.80, 3.47)	4.33×10^{-10}	2.16 (0.43, 3.89);	1.42×10^{-2}
**Low/Other**	<16	-1.05 (-2.03, -0.08)	3.82×10^{-2}	-1.18 (-3.15, 0.79);	0.24
	26–35	1.22 (1.12, 1.32)	$<1 \times 10^{-16}$	0.97 (0.76, 1.18)	$<1 \times 10^{-16}$
	≥36	3.40 (2.98, 3.81)	$<1 \times 10^{-16}$	3.15 (2.36, 3.95)	8.65×10^{-15}
**Cognitive function score**					
**Medium**	<16	-0.86 (-1.20, -0.52)	1.44×10^{-5}	-0.34 (-0.95, 0.28)	0.28
	26–35	0.57 (0.55, 0.59)	$<1 \times 10^{-16}$	-0.25 (-0.29, -0.21)	$<1 \times 10^{-16}$
	≥36	0.62 (0.55, 0.69)	$<1 \times 10^{-16}$	-0.64 (-0.76, -0.53)	$<1 \times 10^{-16}$
**High**	<16	-0.67 (-1.17, -0.17)	2.48×10^{-2}	0.03 (-0.76, 0.82)	0.93
	26–35	0.60 (0.57, 0.64)	$<1 \times 10^{-16}$	-0.21 (-0.28, -0.15)	0.17
	≥36	0.76 (0.65, 0.88)	$<1 \times 10^{-16}$	-0.53 (-0.70, -0.37)	0.48
**Low/Other**	<16	-0.55 (-0.70, -0.40)	0.24	0.22 (-0.05, 0.48)	0.11
	26–35	0.56 (0.54, 0.57)	$<1 \times 10^{-16}$	-0.21 (-0.23, -0.18)	$<1 \times 10^{-16}$
	≥36	0.77 (0.71, 0.83)	$<1 \times 10^{-16}$	-0.44 (-0.53, -0.36)	$<1 \times 10^{-16}$
**Resilience score**					
**Medium**	<16	-0.27 (-0.60, 0.06)	0.32	-0.00 (-0.82, 0.82)	0.10
	26–35	0.33 (0.31, 0.35)	$<1 \times 10^{-16}$	-0.32 (-0.37, -0.27)	$<1 \times 10^{-16}$
	≥36	0.50 (0.42, 0.57)	$<1 \times 10^{-16}$	-0.37 (-0.50, -0.24)	2.09×10^{-8}
**High**	<16	-0.29 (-0.79, 0.20)	0.44	-0.11 (-1.19, 0.97)	0.84
	26–35	0.33 (0.30, 0.37)	$<1 \times 10^{-16}$	-0.33 (-0.40, -0.25)	$<1 \times 10^{-16}$
	≥36	0.50 (0.39, 0.62)	3.34×10^{-6}	-0.55 (-0.74, -0.35)	5.58×10^{-8}
**Low/Other**	<16	-0.34 (-0.48, -0.19)	8.68×10^{-4}	0.14 (-0.14, 0.41)	0.33
	26–35	0.34 (0.33, 0.36)	$<1 \times 10^{-16}$	-0.27 (-0.30, -0.25)	$<1 \times 10^{-16}$
	≥36	0.60 (0.55, 0.66)	$<1 \times 10^{-16}$	-0.37 (-0.46, -0.27)	1.07×10^{-13}

CI, confidence interval

Reference maternal age category: 16–25 years

P-interaction for systolic blood pressure, cognitive function score, resilience score in conventional analyses is 0.183, 0.006, 0.313 from likelihood ratio tests comparing models with and without maternal age × grand paternal socioeconomic index interaction for each outcome.

Table S11. Cohort II - association of maternal age at delivery and type 2 diabetes in adult offspring – stratified by grandparental socioeconomic index

Model type	Maternal age at delivery (years)	Medium (Ref) HR (95% CI), *p* value	High HR (95% CI), *p* value	Low/Other (95% CI), *p* value	*p*-interaction
**Conventional model**	**<16**	1.53 (1.01–2.31), 4.36×10^{-2}	1.19 (0.64–2.22), 0.58	0.98 (0.80–1.21), 0.89	0.61
	**26-35**	0.93 (0.88–0.98), 1.22×10^{-2}	0.93 (0.86–1.02), 0.11	0.92 (0.89–0.96), 5.86×10^{-6}	
	**36+**	1.19 (0.80–1.78), 0.40	1.41 (0.84–2.40), 0.12	1.02 (0.76–1.38), 0.89	
**Sibling-comparison model**	**<16**	0.83 (0.28, 2.45), 0.74	1.07 (0.40, 2.85), 0.89	0.43 (0.27, 0.69), 3.96×10^{-4}	Not applicable
	**26-35**	1.09 (0.98, 1.21), 0.11	1.24 (1.06, 1.46), 6.40×10^{-3}	1.11 (1.05, 1.18), 3.28×10^{-4}	
	**36+**	1.22 (0.65, 2.28), 0.54	3.26 (1.28, 8.30), 1.33×10^{-2}	1.69 (1.07, 2.68), 2.37×10^{-4}	

Ref, reference; HR, hazard ratio; CI, confidence interval
Reference maternal age category: 16–25 years

Table S12. Cohort II - association of maternal age at delivery and adult offspring depression – stratified by grandparental socioeconomic index

Model type	Maternal age at delivery	Medium (Ref) HR (95% CI), *p* value	High HR (95% CI), *p* value	Low/Other HR (95% CI), *p* value	*p*-interaction
**Conventional model**	**<16**	1.14 (0.95–1.37), 0.16	1.00 (0.75–1.32), 0.98	0.90 (0.82–0.99), 2.83 × 10^{-2}	1.92 × 10^{-9}
	**26-35**	1.14 (1.13–1.16), <1 × 10^{-16}	1.16 (1.13–1.18), <1 × 10^{-16}	1.20 (1.19–1.21), <1 × 10^{-16}	
	**36+**	1.57 (1.52–1.62), <1 × 10^{-16}	1.57 (1.50–1.66), <1 × 10^{-16}	1.68 (1.64–1.72), <1 × 10^{-16}	
**Sibling-comparison model**	**<16**	0.80 (0.56, 1.15), 0.24	0.88 (0.56, 1.38), 0.58	0.56 (0.48, 0.66), 8.19 × 10^{-13}	Not applicable
	**26-35**	1.69 (1.65, 1.74), <1 × 10^{-16}	1.80 (1.73, 1.88), <1 × 10^{-16}	1.83 (1.80, 1.86), <1 × 10^{-16}	
	**36+**	3.25 (3.05, 3.45), <1 × 10^{-16}	3.29 (2.98, 3.63), <1 × 10^{-16}	3.70 (3.54, 3.86), <1 × 10^{-16}	

Ref, reference; HR, hazard ratio; CI, confidence interval
Reference maternal age category: 16–25 years

Table S13. Cohort II - association of maternal age at delivery and adult offspring anxiety – stratified by grandparental socioeconomic index

Model type	Maternal age at delivery	Medium (Ref) HR (95% CI)	High HR (95% CI)	Low/Other (95% CI), *p* value	P-interaction
**Conventional model**	**<16**	0.90 (0.73–1.11), 0.32	1.11 (0.84–1.46), 0.45	0.85 (0.77–0.94), 1.45 × 10^{-3}	<0.001
	**26-35**	1.21 (1.19–1.22), <1 × 10^{-16}	1.23 (1.20–1.26), <1 × 10^{-16}	1.27 (1.26–1.28), <1 × 10^{-16}	
	**36+**	1.71 (1.66–1.77), <1 × 10^{-16}	1.75 (1.66–1.84); <1 × 10^{-16}	1.85 (1.80–1.89), <1 × 10^{-16}	
**Sibling-comparison model**	**<16**	0.55 (0.37, 0.84), 5.10 × 10^{-3}	0.50 (0.29, 0.85), 1.02 × 10^{-2}	0.51 (0.43, 0.60), 1.77 × 10^{-14}	Not applicable
	**26-35**	1.83 (1.78, 1.88), <1 × 10^{-16}	2.00 (1.91, 2.10), <1 × 10^{-16}	1.94 (1.91, 1.98), <1 × 10^{-16}	
	**36+**	3.90 (3.66, 4.17), <1 × 10^{-16}	3.90 (3.52, 4.32), <1 × 10^{-16}	4.55 (4.34, 4.77), <1 × 10^{-16}	

Ref, reference; HR, hazard ratio; CI, confidence interval
Reference maternal age category: 16–25 years

Table S14. Cohort I - association of maternal age at delivery and body mass index of offspring at conscription – effect modification by birth cohort

Cohort	Maternal age at delivery (years)	Conventional model RRR (95% CI)	*p* value	*p*-interaction	Sibling-comparison model RRR (95% CI)	*p* value	*p*-interaction
**Underweight**							
**Early** [a]	**<16**	0.66 (0.42, 1.02)	0.60	0.047	0.71 (0.59, 0.85)	1.73×10^{-4}	$<1 \times 10^{-16}$
	**26–35**	0.94 (0.84, 1.06)	0.36		1.06 (1.04, 1.08)	8.12×10^{-10}	
	**≥36**	0.97 (0.92, 1.02)	0.25		0.86 (0.80, 0.93)	5.40×10^{-4}	
**Late** [a]	**<16**	1.23 (0.64, 2.35)	0.53		0.78 (0.55, 1.12)	0.17	
	**26–35**	1.02 (0.98, 1.05)	0.38		1.19 (1.17, 1.22)	$<1 \times 10^{-16}$	
	**≥36**	0.94 (0.84, 1.05)	0.25		1.14 (1.10, 1.18)	2.51×10^{-14}	
**Overweight/Obese**							
**Early** [a]	**<16**	1.20 (0.96, 1.51)	0.12	0.047	0.48 (0.35, 0.67)	1.08×10^{-5}	$<1 \times 10^{-16}$
	**26–35**	0.96 (0.89, 1.05)	0.39		1.00 (0.97, 1.03)	0.83	
	**≥36**	0.95 (0.92, 0.98)	8.70×10^{-4}		0.81 (0.72, 0.91)	3.61×10^{-4}	
**Late** [a]	**<16**	1.31 (0.91, 1.89)	0.15		0.99 (0.51, 1.93)	0.99	
	**26–35**	0.87 (0.86, 0.89)	$<1 \times 10^{-16}$		1.22 (1.17, 1.26)	$<1 \times 10^{-16}$	
	**≥36**	0.90 (0.85, 0.96)	8.70×10^{-4}		1.23 (1.15, 1.31)	9.62×10^{-11}	

RRR, relative risk ratio; CI, confidence interval
Reference maternal age category: 16–25 years; Body mass index reference category: normal weight

[a] Birth cohort before median birth year of the sample is considered early and the one born during and later than the median birth year is considered late

Table S15. Cohort I - association of maternal age at delivery and body mass index of offspring at conscription – effect modification by birth cohort

Cohort	Maternal age at delivery (years)	Conventional model β (95% CI)	*p* value	*p*-interaction	Sibling-comparison model β (95% CI)	*p* value	*p*-interaction
**Systolic blood pressure**							
**Early** [a]	**<16**	-0.48 (-0.64, -0.33)	1.74×10^{-9}	0.67	0.09 (-0.09, 0.27)	0.390	0.17
	**26–35**	0.49 (0.43, 0.54)	$<1 \times 10^{-16}$		-0.25 (-0.27, -0.24)	$<1 \times 10^{-16}$	
	**≥36**	0.56 (0.52, 0.60)	$<1 \times 10^{-16}$		-0.51 (-0.58, -0.45)	$<1 \times 10^{-16}$	
**Late** [a]	**<16**	-0.78 (-1.05, -0.51)	1.74×10^{-8}		0.16 (-0.49, 0.80)	0.63	
	**26–35**	0.47 (0.46, 0.49)	$<1 \times 10^{-16}$		-0.20 (-0.23, -0.18)	$<1 \times 10^{-16}$	
	**≥36**	0.56 (0.52, 0.60)	$<1 \times 10^{-16}$		-0.35 (-0.39, -0.31)	$<1 \times 10^{-16}$	
**Cognitive function scores**							
**Early** [a]	**<16**	-0.25 (-0.41, -0.10)	1.36×10^{-3}	1.22×10^{-2}	0.09 (-0.10, 0.28)	0.36	0.01
	**26–35**	0.21 (0.16, 0.26)	$<1 \times 10^{-16}$		-0.19 (-0.21, -0.18)	$<1 \times 10^{-16}$	
	**≥36**	0.38 (0.34, 0.42)	$<1 \times 10^{-16}$		-0.34 (-0.41, -0.27)	$<1 \times 10^{-16}$	
**Late** [a]	**<16**	-0.51 (-0.80, -0.22)	5.17×10^{-4}		-0.37 (-1.05, 0.31)	0.29	
	**26–35**	0.28 (0.27, 0.29)	$<1 \times 10^{-16}$		-0.21 (-0.24, -0.18)	$<1 \times 10^{-16}$	
	**≥36**	0.38 (0.34, 0.42)	$<1 \times 10^{-16}$		-0.35 (-0.39, -0.30)	$<1 \times 10^{-16}$	
**Resilience scores**							
**Early** [a]	**<16**	-0.88 (-1.84, 0.07)	7.06×10^{-2}	0.33	-1.32 (-2.54, -0.09)	3.54×10^{-2}	0.33
	**26–35**	0.65 (0.34, 0.96)	4.29×10^{-5}		0.35 (0.23, 0.46)	3.39×10^{-9}	
	**≥36**	2.88 (2.57, 3.19)	$<1 \times 10^{-16}$		0.47 (0.00, 0.93	4.10×10^{-2}	
**Late** [a]	**<16**	-0.97 (-2.86, 0.92)	0.32		-7.31 (-13.24, -1.37)	1.58×10^{-2}	
	**26–35**	0.90 (0.80, 0.99)	$<1 \times 10^{-16}$		1.26 (1.03, 1.48)	$<1 \times 10^{-16}$	
	**≥36**	2.88 (2.57, 3.19)	$<1 \times 10^{-16}$		2.49 (2.14, 2.85)	$<1 \times 10^{-16}$	

CI, confidence interval
Reference maternal age category: 16–25 years

[a] Birth cohort before median birth year of the sample is considered early and the one born during and later than the median birth year is considered late

Table S16. Cohort II - association of maternal age at delivery and type 2 diabetes in adult offspring– effect modification by birth cohort

Model type	Maternal age at birth	Early [a] HR (95% CI), *p* value	Late [a] HR (95% CI), *p* value	P-interaction
**Conventional model**	**<16**	0.95 (0.77–1.17), 0.64	1.91 (1.34–2.74), 3.97×10^{-4}	8.05×10^{-3}
	**26-35**	0.88 (0.74–1.05), 0.16	0.90 (0.87–0.93), 2.86×10^{-11}	
	**36+**	1.09 (0.88–1.36), 0.44	NA (NA–NA)	
**Sibling-comparison model**	**<16**	0.73 (0.59–0.91), 4.48×10^{-3}	0.40 (0.08–1.89), 0.25	8.33×10^{-6}
	**26-35**	1.27 (1.23–1.30), $<1 \times 10^{-16}$	1.15 (1.08–1.21), 3.24×10^{-6}	
	**36+**	2.42 (2.14–2.74), $<1 \times 10^{-16}$	1.63 (1.46–1.81), $<1 \times 10^{-16}$	

HR, hazard ratio; CI, confidence interval
Reference maternal age category: 16–25 years, refence birth cohort: early

[a] Birth cohort before median birth year of the sample is considered early and the one born during and later than the median birth year is considered late

Table S17. Cohort II - association of maternal age at delivery and depression in adult offspring– effect modification by birth cohort

Model type	Maternal age at birth	Early [a] HR (95% CI), *p* value	Late [a] HR (95% CI), *p* value	P-interaction
**Conventional model**	**<16**	1.09 (1.00–1.19), 0.06	1.32 (1.10–1.57), 2.15×10^{-3}	$< 1 \times 10^{-16}$
	**26-35**	1.08 (1.06–1.10), $<1 \times 10^{-16}$	0.88 (0.87–0.89), $<1 \times 10^{-16}$	
	**36+**	1.14 (1.12–1.16); $<1 \times 10^{-16}$	NA (NA–NA)	
**Sibling-comparison model**	**<16**	0.68 (0.61–0.77), 5.20×10^{-3}	0.78 (0.53–1.14), 0.20	$< 1 \times 10^{-16}$
	**26-35**	1.78 (1.76–1.80), $<1 \times 10^{-16}$	1.48 (1.46–1.51), $<1 \times 10^{-16}$	
	**36+**	4.59 (4.39–4.79), $<1 \times 10^{-16}$	2.81 (2.72–2.90), $<1 \times 10^{-16}$	

HR, hazard ratio; CI, confidence interval
Reference maternal age category: 16–25 years, refence birth cohort: early

[a] Birth cohort before median birth year of the sample is considered early and the one born during and later than the median birth year is considered late

Table S18. Cohort II - association of maternal age at delivery and anxiety in adult offspring– effect modification by birth cohort

Model type	Maternal age at birth	Early [a] HR (95% CI), *p* value	Late [a] HR (95% CI), *p* value	P-interaction
**Conventional model**	**<16**	1.00 (0.91–1.11); 0.96	1.33 (1.11–1.60), 1.81×10^{-3}	$< 1 \times 10^{-16}$
	**26-35**	1.13 (1.12–1.15); $<1 \times 10^{-16}$	0.91 (0.90–0.92), $<1 \times 10^{-16}$	
	**36+**	1.22 (1.20–1.24); <1 × 10⁻16		
**Sibling-comparison model**	**<16**	0.59 (0.52–0.68); $<1 \times 10^{-16}$	0.78 (0.54–1.14), 0.21	$< 1 \times 10^{-16}$
	**26-35**	1.93 (1.91–1.96); $<1 \times 10^{-16}$	1.54 (1.51–1.57), $<1 \times 10^{-16}$	
	**36+**	6.01 (5.72–6.32); $<1 \times 10^{-16}$	3.29 (3.18–3.40), $<1 \times 10^{-16}$	

HR, hazard ratio; CI, confidence interval
Reference maternal age category: 16–25 years, refence birth cohort: early

[a] Birth cohort before median birth year of the sample is considered early and the one born during and later than the median birth year is considered late

Table S19. Cohort II - association of maternal age at delivery and type 2 diabetes in adult offspring– effect modification by sex

Model type	Maternal age at birth	Women HR (95% CI), *p* value	Men HR (95% CI), *p* value	P-interaction
**Conventional model**	**<16**	0.97 (0.74–1.28), 0.83	1.16 (0.92–1.47), 0.22	1.40×10^{-6}
	**26-35**	0.99 (0.95–1.03), 0.69	0.87 (0.84–0.90), 1.02×10^{-13}	
	**36+**	1.45 (1.09–1.92), 9.93×10^{-3}	0.83 (0.59–1.18); 0.31	
**Sibling-comparison model**	**<16**	0.65 (0.47–0.89), 4.81×10^{-3}	0.79 (0.59–1.07); 0.13	1.93×10^{-3}
	**26-35**	1.30 (1.26–1.34), $<1 \times 10^{-16}$	1.21 (1.18–1.25); $<1 \times 10^{-16}$	
	**36+**	2.11 (1.90–2.34), $<1 \times 10^{-16}$	1.86 (1.68–2.06); $<1 \times 10^{-16}$	

HR, hazard ratio; CI, confidence interval
Reference maternal age category: 16–25 years, refence sex: women

Table S20. Cohort II - association of maternal age at delivery and depression in adult offspring– effect modification by sex

Model type	Maternal age at birth	Women HR (95% CI), *p* value	Men HR (95% CI), *p* value	P-interaction
Conventional model	<16	0.89 (0.80–0.99), 0.03	1.02 (0.91–1.16), 0.71	0.045
	26-35	1.19 (1.18–1.20), <1 × 10^{-16}	1.17 (1.16–1.18), <1 × 10^{-16}	
	36+	1.64 (1.60–1.68); <1 × 10^{-16}	1.62 (1.58–1.67), <1 × 10^{-16}	
Sibling-comparison model	<16	0.60 (0.52–0.70), 1.38 × 10^{-13}	0.67 (0.58–0.79), 6.87 × 10^{-7}	4.00 × 10^{-7}
	26-35	1.89 (1.87–1.92), <1 × 10^{-16}	1.81 (1.78–1.83), <1 × 10^{-16}	
	36+	4.56 (4.43–4.69), <1 × 10^{-16}	4.33 (4.19–4.46), <1 × 10^{-16}	

HR, hazard ratio; CI, confidence interval
Reference maternal age category: 16–25 years, refence sex: women

Table S21. Cohort II - association of maternal age at delivery and anxiety in adult offspring– effect modification by sex

Model type	Maternal age at birth	Women HR (95% CI), *p* value	Men HR (95% CI), *p* value	P-interaction
Conventional model	<16	0.80 (0.71–0.90); 1.60 × 10^{-4}	0.97 (0.85–1.10), 0.66	2.28 × 10^{-11}
	26-35	1.29 (1.27–1.30), <1 × 10^{-16}	1.23 (1.21–1.24), <1 × 10^{-16}	
	36+	1.88 (1.84–1.92), <1 × 10^{-16}	1.73 (1.68–1.78), <1 × 10^{-16}	
Sibling-comparison model	<16	0.50 (0.43–0.59), <1 × 10^{-16}	0.61 (0.52–0.72), 6.14 × 10^{-9}	2.28 × 10^{-11}
	26-35	2.07 (2.04–2.10), <1 × 10^{-16}	1.93 (1.91–1.96), <1 × 10^{-16}	
	36+	5.87 (5.68–6.06), <1 × 10^{-16}	5.36 (5.18–5.54), <1 × 10^{-16}	

HR, hazard ratio; CI, confidence interval
Reference maternal age category: 16–25 years, refence sex: women

References

1. Ludvigsson JF, Berglind D, Sundquist K, et al. The Swedish military conscription register: opportunities for its use in medical research. Eur J Epidemiol 2022;37:767-77. https://doi.org/10.1007/s10654-022-00887-0
2. CARLSTEDT B, GUSTAFSSON J-E. Construct validation of the Swedish Scholastic Aptitude Test by means of the Swedish Enlistment Battery. Scandinavian Journal of Psychology 2005;46:31-42. https://doi.org/https://doi.org/10.1111/j.1467-9450.2005.00432.x
3. Carlsted B, MÅRdberg B. Construct Validity of the Swedish Enlistment Battery. Scandinavian Journal of Psychology 2008;34:353-62. https://doi.org/10.1111/j.1467-9450.1993.tb01131.x
4. Nilsson PM, Nyberg P, Östergren P-O. Increased susceptibility to stress at a psychological assessment of stress tolerance is associated with impaired fetal growth. International Journal of Epidemiology 2001;30:75-80. https://doi.org/10.1093/ije/30.1.75
5. Ludvigsson JF, Andersson E, Ekbom A, et al. External review and validation of the Swedish national inpatient register. BMC Public Health 2011;11:450. https://doi.org/10.1186/1471-2458-11-450
6. Wettermark B, Hammar N, Fored CM, et al. The new Swedish Prescribed Drug Register--opportunities for pharmacoepidemiological research and experience from the first six months. Pharmacoepidemiol Drug Saf 2007;16:726-35. https://doi.org/10.1002/pds.1294
7. Ludvigsson JF, Svedberg P, Olén O, et al. The longitudinal integrated database for health insurance and labour market studies (LISA) and its use in medical research. European Journal of Epidemiology 2019;34:423-37. https://doi.org/10.1007/s10654-019-00511-8
8. Aizer A, Devereux PJ, Salvanes KG. Grandparents, Moms, or Dads? Why Children of Teen Mothers Do Worse in Life. National Bureau of Economic Research Working Paper Series No. 25165. Cambridge, MA: National Bureau of Economic Research; 2018. https://www.nber.org/papers/w25165 (4 July 2026, date last accessed).
9. Hernández-Díaz S, Schisterman EF, Hernán MA. The birth weight "paradox" uncovered? Am J Epidemiol 2006;164:1115-20. https://doi.org/10.1093/aje/kwj275
10. Hertzman C, Power C. Health and human development: understandings from life-course research. In: ed.^eds. The biological and social determinants of child development. 1 ed. London: Psychology Press, 2003, 26.
11. Sundrani DP, Roy SS, Jadhav AT, et al. Sex-specific differences and developmental programming for diseases in later life. Reproduction Fertility and Development 2017;29:2085-99. https://doi.org/10.1071/rd16265
12. Singh S, Darroch JE. Adolescent Pregnancy and Childbearing: Levels and Trends in Developed Countries. Family Planning Perspectives 2000;32:14-23. https://doi.org/10.2307/2648144
